# Employment preferences of healthcare workers in South Africa: Findings from a discrete choice experiment

**DOI:** 10.1371/journal.pone.0250652

**Published:** 2021-04-28

**Authors:** Alexandra Mumbauer, Michael Strauss, Gavin George, Phuti Ngwepe, Charl Bezuidenhout, Lindsey de Vos, Andrew Medina-Marino

**Affiliations:** 1 Qode Health Solutions, Pretoria, South Africa; 2 Health Economics and HIV and AIDS Research Division (HEARD), University of KwaZulu-Natal, Durban, South Africa; 3 Research Unit, Foundation for Professional Development, East London, Eastern Cape, South Africa; 4 Desmond Tutu HIV Centre, University of Cape Town, Cape Town, South Africa; 5 Perelman School of Medicine, University of Pennsylvania, Philadelphia, Pennsylvania, United States of America; University of Witwatersrand/NHLS, SOUTH AFRICA

## Abstract

There is a maldistribution of human resources for health globally, with many Lower- and Middle-Income Countries experiencing significant shortages. We examined healthcare workers’ job preferences in South Africa to identify factors which potentially influence employment decisions. A discrete choice experiment was conducted among 855 South African healthcare workers critical to its national HIV testing and treatment programs. Job characteristics included workload, workplace culture, availability of equipment, training opportunities, sector and facility type, location, salary and benefits. Main effects analysis was conducted using fixed effects logistic regression. Interaction effects identified divergence in preferences. Heavy workload (OR = 0.78; 95% C.I. 0.74–0.83), poor workplace culture (odds ratio 0.66; 95% C.I. 0.62–0.69), insufficient availability of equipment (OR = 0.67; 95% C.I. 0.63–0.70) and infrequent training opportunities (OR = 0.75; 95% C.I. 0.71–0.80) had large, significant effects on worker preferences. An increase in salary of 20% (OR = 1.29; 95% C.I. 1.16–1.44) had a positive effect on preferences, while a salary decrease of 20% (OR = 0.55; 95% C.I. 0.49–0.60) had a strong negative effect. Benefits packages had large positive effects on preferences: respondents were twice as likely to choose a job that included medical aid, pension and housing contributions worth 40% of salary (OR = 2.06; 95% C.I. 1.87–2.26), holding all else constant. Although salary was important across all cadres, benefits packages had larger effects on job preferences than equivalent salary increases. Improving working conditions is critical to attracting and retaining appropriate health cadres responsible for the country’s HIV services, especially in the public sector and underserved, often rural, communities. Crucially, our evidence suggests that factors amenable to improvement such as workplace conditions and remuneration packages have a greater influence on healthcare workers employment decisions than employment sector or location.

## Introduction

There is a maldistribution of human resources for health (HRH) globally with many Lower- and Middle-Income Countries (LMIC) possessing significant shortages, leading to fragile public health systems [1]. Within countries disparities remain in the distribution of HRH, with urban centers exhibiting higher ratios of health workers to population compared to rural areas [2]. Additionally, the private health sector has often been perceived as attracting health workers away from the public health sector [3]. These dynamics are particularly apparent in South Africa, as the country possesses parallel and unequal health systems: A private, for-profit system financed primarily by those who can afford health insurance, and a government-financed public system that provides care for the vast majority of the population.

In South Africa, though approximately 40% of general practitioners and nurses work in the private sector [4], they only provide services to those with private health insurance, which accounts for approximately 17% of the population [5]. Health personnel frequently migrate between public and private sectors and between facilities in urban and rural settings or move abroad. Previous studies have highlighted various push and pull factors that drive this migration, including work-related stress linked to heavy workloads, remuneration and availability of opportunities for development [6]. Many healthcare workers have negative perceptions of the public health system, characterized by high patient loads, long working hours, inadequate resources and occupational hazards, all of which are cited as reasons for leaving or avoiding employment in this sector [7]. However, government initiatives, such as the introduction of the Occupation Specific Dispensation, an official list of salary structures that are unique to each identified occupation in the public service in South Africa, has reduced the pay gap between the public and private sector [8].

Understanding employment decisions of healthcare workers is critical for the South African government to implement strategies aimed at adequately resourcing the public health system, sustain its HIV program and transition to a national health insurance (NHI) system [9]. In addition, public facilities, especially primary health clinics and rural facilities, will need the right cadres of healthcare workers to sustain their services, including those that contribute to the country’s HIV response [10–13]. A study conducted amongst health practitioners working in the private health sector in South Africa has highlighted that respondents held negative views on the impending NHI, with 20.8% of those surveyed indicating that they had already taken steps to emigrate, whilst a further 41.6% suggesting that emigration was likely when the NHI was implemented [14]. Efforts to strengthen public health systems therefore have to identify and consider employment preferences if programs aimed at attracting and retention of healthcare workers are to succeed. Ultimately, a better understanding of the trade-offs healthcare workers make when applying for and considering jobs could guide the development of policies to attract and retain public sector personnel, especially in under-resourced and under-served areas [15–17].

Previous discrete choice experiments (DCEs) undertaken with healthcare workers in South Africa found preferences relating to salary and remuneration packages, as well as facility infrastructure and management, influenced employment considerations [15, 18, 19]. Additionally, non-financial factors such as the availability of training and development opportunities were found to be important factors in a study of nurses who had recently completed their training and were entering the job market for the first time [15]. While informative, these studies only included nurses or nursing students and did not include other key healthcare workers or those from the private sector. Furthermore, while Blaauw and colleagues [15] investigated the effectiveness of different policies on attracting nurses to rural areas, none of these studies explored factors associated with migration between urban and rural areas or between the public and private sectors. Finally, none of these studies involved healthcare workers who provided direct HIV services or contexualized their findings within the on-going scale-up and sustainability of South Africa’s national HIV treatment program, which is the largest in the world; Penn-Kekana and colleagues [19] focused on maternal health services only. Given these major limitations and South Africa’s transition to a national health insurance system, we conduced a study amongst multiple cadres of healthcare workers in both the public and private sectors to identify employment preferences that would attract healthcare workers to work in under-served and under-resourced areas.

## Materials and methods

This study was embedded within a larger endeavor to provide the South African National Department of Health actionable information to achieve its Human Resources for Health 2030 Vision, which includes having adequate and appropriate human resources for expanding HIV services [20].

### Discrete choice experiment tool design

The study team agreed in advance to limit the final number of attributes in the DCE design to a maximum of eight, aligned to guidance from literature to ensure that participants are able to consider all attributes when making a choice [21, 22]. We conducted a literature review on healthcare worker retention and employment preferences in South Africa to generate a list of potential attributes and levels for choice sets. Through the literature review, a list of 12 potential attributes were identified: 1) quality and variety of specialty training; 2) availability of good jobs; 3) prospects for professional advancement; 4) infrastructure/availability of equipment; 5) salary; 6) better working conditions; 7) workplace culture/management; 8) job security; 9) location; 10) benefits; 11) allowances; and 12) workload. A sub-set of participants in the larger activity associated with this study who were healthcare workers employed in either the public or private sectors were included in a brief survey to confirm that potential attributes identified through literature review were important to the study population. Respondents who indicated that they had previously changed sector of employment (n = 122) were asked to rate each potential attribute based on the level of influence each attribute had on their decision to move sectors. Respondents who indicated that they had previously considered moving between employment sectors (n = 120) were asked to rate each potential attribute based on the level of influence each attribute had on their intention to move sectors. A small sample (n = 6) of in-depth interviews (IDIs) with healthcare workers participating in the larger activity associated with this study were also analysed to refine attribute selection and to clarify the levels of the attributes that should be included. Data were included from all participants of the larger activity who had completed the survey or IDI by the time that attributes and levels were being finalised.

Table 1 details the final list of eight attributes and levels included in the DCE design. Findings from the survey and IDIs suggested that availability of good jobs and job security were not as important as the other attributes to the study population and were excluded from the final list of attributes. Since allowances form part of benefits packages for healthcare workers in South Africa, an allowances attribute was not included in addition to the benefits attribute. Because professional advancement for healthcare workers is often dependent on opportunities for training, the attribute “prospects for professional advancement” was potentially redundant and thus excluded.

**Table 1:** Attributes and levels included in the DCE design.

Attribute	Level 1 (Baseline)	Level 2	Level 3	Level 4
**Workload**	Manageable	Heavy	-	-
**Workplace Culture**	Enabling(“Good”) culture	Poor culture	-	-
**Availability of Equipment**	Sufficient	Insufficient	-	-
**Opportunities for Training**	Frequent	Infrequent/minimal	-	-
**Sector and Facility type**	Public clinic	Public Hospital	Private clinic	Private Hospital
**Location**	Urban; No relocation	Urban; Relocation	Rural; No relocation	Rural; Relocation
**Salary**	5% increase on current salary	10% increase on current salary	20% increase on current salary	Salary cut of 20%
**Benefits**	None	Medical aid contribution to the value of 7% of salary	Medical aid and pension contribution of 20% of salary	Medical aid, pension and housing contribution of 40% of salary

Note: The column “Level 1” shows the baseline scenario and the level for each attribute that was used as a reference category in the analysis (see section 2.4). Four attributes had only two levels each, and four attributes had four levels each. “Level 2”, “Level 3” and “Level 4” are generic column headings

A binary design with no “opt-out” was used for this study to maximize the amount of information collected from each respondent. Although the primary characteristic of interest was the choice between the public and private sector, this study used an unlabelled design (including public/private sector as an attribute rather than an alternative specific label) to reduce the likelihood of participants inferring too much additional information about the type of job or overemphasizing the importance of this particular characteristic in relation to other attributes. Given the fairly large number of attributes and levels, an orthogonal main effects plan (OMEP) was used to generate a fractional factorial design with 32 statistically representative choice sets (from a total of 4096) using SPSS23. These profiles were used as the first alternative in each choice set and were generated to be orthogonal and ensure level balance [23]. In order to optimize the D-efficiency of the design, the second alternative in each choice set was generated by systematically and cyclically adding one level to each attribute in the first choice set [24]. The 32 choice sets were divided into four versions of eight choice sets each using a blocking variable, which was included in the OMEP, to ensure that design characteristics were preserved in each version [24]. The final design resulted in a D-efficiency of 95.63%. Respondents within each province and cadre were randomly assigned to one of the four versions of the eight choice sets.

### Study population and sampling

We recruited healthcare workers providing direct or indirect HIV care and treatment services from Gauteng, Limpopo, and Mpumalanga provinces, which were selected for their mix of urban and rural settings and availability of public and private healthcare services. Whilst Gauteng contains three of South Africa’s eight major metropolitan areas and has the highest proportion of residents who use private healthcare services, Mpumalanga and Limpopo are predominantly rural and have relatively low uptake of private healthcare services. Healthcare worker cadres included general practitioners, professional nurses, support nurses (enrolled nurses and enrolled nursing assistants), and pharmacy personnel (pharmacists and pharmacy assistants) working in the public, private, or non-governmental organization (NGO) sector. Participants were introduced to the study and invited to participate through an email distribution mailing list (“electronic recruitment”) or through the study team at healthcare facilities (“in-person recruitment”) following buy-in meetings with facility management. All participants were introduced to the study with a short narrative on the DCE process followed by a detailed definition of each attribute and accompanying attribute levels (see electronic supplementary material). These were read aloud by study staff for participants completing the questionnaire in-person and presented on the screen for participants completing the questionnaire electronically. Each attribute was introduced to the participant separately, and the participant had to indicate that they understood the attribute before moving onto the next. One DCE question was repeated across all versions to check for consistency in responses to the in-person and electronic questionnaires, followed by the eight unique choice sets in the version to which they were assigned (see electronic supplementary material for an example of a choice set).

Access to healthcare workers’ professional details for the electronic distribution of surveys were obtained from the Foundation for Professional Development alumni databases and collaborating public and private institutions. In-person recruitment was limited to Tshwane District (Gauteng), Capricorn District (Limpopo), and Nkangala District (Mpumalanga). These districts were chosen because they each contain an urban area with a high concentration of public and private healthcare workers that the study could recruit from.

For sample size, Orme [25] suggests a minimum of n participants for a DCE design where L is the maximum number of levels in any attribute (four), S is the number of alternatives in each choice set (two) and J is the number of choice sets from the design that are presented to each respondent (eight), so that:

$$n=500\frac{L}{SJ}$$

Thus, a minimum sample size of 125 respondents was needed per unique stratification used in the analysis. Healthcare workers were selected as evenly as possible per cadre, province and sector, across the full sample of 855 respondents (see Results).

### Data analysis

Analysis was conducted using STATA 13.0 [Stata Corp, College Station TX]. A fixed effects logit model, using dummy coding, was used to estimate parameters. Results are presented as odds ratios in comparison to a baseline scenario (Table 1). We used fixed effect logit models, which are commonly used in health DCEs [26–29], under the assumptions of independent and identically distributed error terms and the independence of irrelevant alternatives (IIA). In this study design, choice set options represent two different job profiles, meaning that violations of the IIA assumption are unlikely and that a fixed effects model is appropriate. As a further check, fixed effect logit estimates were compared to random effects logit estimates, and a Hausman test was conducted to test for violations of the IIA assumptions [30]. The Hausman test statistic returned a value of -833.35, which should be taken as further evidence that the IIA assumption holds [30].

The fixed logit model estimates the probability of choosing one alternative over another as follows:

$${Pr}_{ik}=\frac{exp({\beta X}_{ik})}{\sum_{j=1}^{J}exp({\beta X}_{ij})},\text{for}\;\text{all}\;\text{alternatives}\;\text{J}\;\text{in}\;\text{the}\;\text{choice}\;\text{set}$$

where *Pr*_*ik*_ is the probability of individual *i* choosing alternative *k* from a set of alternatives *J*. ***β*** is a column vector of parameter estimates associated with ***X***_*ik*_, which is a row vector of the levels of the attributes in alternative *k* chosen by individual *I* [31].

All effects are reported as odds ratios, compared to baseline levels for each attribute, which are shown in brackets next to the attribute level. To test how healthcare worker cadre influenced preferences, dummy variables were generated for each cadre. Interaction terms were created by multiplying these dummy variables with attribute level dummy variables for inclusion in the model [32]. Where interaction models indicated significant differences between groups, separate stratified models were run to describe the preferences of each group.

### Ethics

Ethics approval was granted by the Foundation for Professional Development Research Ethics Committee (Registration No. REC-03711-033-RA), South Africa, prior to protocol initiation. Approval to conduct interviews with healthcare workers in public health facilities was obtained from departments of health in Tshwane, Nkangala, and Capricorn districts. Approval to conduct interviews with healthcare workers in private facilities was provided by facility management. All participants provided written informed consent prior to initiating any data collection activities.

## Results

### Demographic characteristics

Of the 855 respondents recruited in this study, 608 were female (71%). The largest number of participants were from Gauteng Province (n = 331; 39%) followed by Limpopo (n = 248; 29%) and Mpumalanga provinces (n = 186; 22%). Half of the participants were from the public sector (n = 431; 51.01%), and a third of all participants were professional nurses (n = 280; 32.75%). A total of 340 respondents (40%) reported having ever moved between sectors; of these, 198 (59%) reported moving from the public sector to the private sector, 85 (25%) from the private sector to the public sector and all others between the NGO and public/private sectors (Table 2).

**Table 2:** Participant socio-demographic and employment characteristics.

	Number	%
**Sex**		
Female	608	71.45
Male	243	28.55
**Marital Status**		
Single	288	33.72
Married	473	55.39
Living Together	31	3.63
Divorced/Separated/Widow	62	7.27
**Province**		
Gauteng	331	38.71
Mpumalanga	186	21.75
Limpopo	248	29.01
Other	90	10.53
**Cadre**		
General Practitioner	254	29.71
Pharmacy Personnel	153	17.89
Professional Nurse	280	32.75
Support Nurses	168	19.65
**Sector Currently Working**		
Private	373	44.11
Public	431	51.01
NGO/NPO	41	4.85
**Type of Facility**		
Primary Health Clinic	168	19.70
Community Health Centre	107	12.54
Hospital	368	43.14
Private Practice	129	15.12
Other	81	9.50
**Location of Current Place of Work**		
Urban	431	50.65
Rural	207	24.32
Peri-Urban	213	25.03
**Movement between sectors**		
Ever moved sectors	340	39.91
Never moved sectors	512	60.09
**Response Type**		
In-Person Questionnaire	436	50.99
Electronic Questionnaire	419	49.01

### Electronic and in-person responses

Almost half of the participants completed the electronic questionnaire (49.10%). The majority of general practitioners (88.98%) responded electronically, while the majority of support nurses responded in person (94.05%) (Table 3). Analysis of the question included for consistency testing showed that there was no difference in the response between participants who completed the electronic questionnaire versus the in-person questionnaire (Pearson’s Chi-square test, p = 0.142).

**Table 3:** Response type by cadre.

	In-Person Questionnaire		Electronic Questionnaire	
	Number	%	Number	%
**General Practitioner**	28	11.02	226	88.98
**Pharmacy Personnel**	90	58.82	63	41.18
**Professional Nurse**	160	57.14	120	42.86
**Support Nurses**	158	94.05	10	5.95
**Total**	436	50.99	419	49.01

### Main effects

Heavy workload (odds ratio 0.78; 95% C.I. 0.74–0.83), poor workplace culture (odds ratio 0.66; 95% C.I. 0.62–0.69), insufficient availability or quality of equipment (odds ratio 0.67; 95% C.I. 0.63–0.70) and infrequent training opportunities (odds ratio 0.75; 95% C.I. 0.71–0.80) had large, significant effects on choice selection (Table 4). Sector and facility type were found to have smaller, though statistically significant, effects on attribute preferences. Respondents were less likely to select a job profile which included a public hospital (odds ratio 0.91; 95% C.I. 0.83–0.99) or private clinic (odds ratio 0.89; 95% C.I. 0.80–0.99) than a choice set which included a public clinic. Where other conditions remained constant, there was no difference in selection preferences between a private hospital and public clinic.

**Table 4:** Main effects fixed logit model.

ATTRIBUTE	Attribute level (Baseline)	Odds Ratio	Std. Error	P-value	95% C.I.	
**WORKLOAD**	Heavy workload (vs manageable workload)**	0.78	0.021	<0.001	0.74	0.83
**WORKPLACE CULTURE**	Poor culture (vs enabling culture)**	0.66	0.018	<0.001	0.62	0.69
**EQUIPMENT**	Insufficient equipment (vs sufficient equipment)**	0.67	0.018	<0.001	0.63	0.70
**TRAINING**	Infrequent training (vs frequent training)**	0.75	0.021	<0.001	0.71	0.80
**SECTOR/ FACILITY**	Public hospital (vs public clinic)*	0.91	0.043	0.042	0.83	0.99
	Private clinic (vs public clinic)*	0.89	0.049	0.036	0.80	0.99
	Private hospital (vs public clinic)	0.99	0.050	0.878	0.90	1.09
**LOCATION**	Urban with relocation (vs urban without relocation)**	0.74	0.036	<0.001	0.68	0.82
	Rural without relocation (vs urban without relocation)*	0.88	0.048	0.020	0.79	0.98
	Rural with relocation (vs urban without relocation)**	0.64	0.030	<0.001	0.58	0.70
**SALARY**	Salary increase of 10% (vs salary increase of 5%)	1.09	0.051	0.063	0.99	1.19
	Salary increase of 20% (vs salary increase of 5%)**	1.29	0.071	<0.001	1.16	1.44
	Salary cut of 20% (vs salary increase of 5%)**	0.55	0.028	<0.001	0.49	0.60
**BENEFITS**	Benefits–medical aid contribution of 7% of salary (vs none)**	1.42	0.067	<0.001	1.29	1.55
	Benefits–pension and medical aid contribution of 20% of salary (vs none)**	1.79	0.098	<0.001	1.61	1.99
	Benefits–pension, medical aid and housing contribution of 40% of salary (vs none)**	2.06	0.097	<0.001	1.87	2.26
		**Number of observations**		**13 604**		
		**Number of groups**		**6 802**		
		**Log Likelihood**		**-4012.8065**		
		**LR Chi**^**2**^**(16)**		**1403.96**		
		**Pseudo R**^**2**^		**0.15**		

*: p<0.05 (Significant);

**: p<0.01 (Highly significant); C.I. = confidence interval; SE = standard error

Compared to the baseline 5% salary increase, an increase in salary of 10% (odds ratio 1.09; 95% C.I. 0.99–1.19) trended toward a positive impact on preferences, while a salary increase of 20% (odds ratio 1.29; 95% C.I. 1.16–1.44) had a significant, positive effect. In comparison, a salary decrease of 20% (odds ratio 0.55; 95% C.I. 0.49–0.60) had the largest negative effect on attribute preferences. Compared to a job profile with no benefits, a job profile with a medical aid contribution worth 7% of total salary (odds ratio 1.42; 95% C.I. 1.29–1.55) showed a strong positive effect. Furthermore, offering a benefits package with medical aid and a pension contribution worth 20% of salary almost doubled the odds of a respondent choosing this alternative (odds ratio 1.79; 95% C.I. 1.61–1.99). The benefits package worth 20% of salary was significantly preferred over increasing salary by 20% (odds ratio 1.29; 95% C.I. 1.16–1.44). Respondents were twice as likely (odds ratio 2.06; 95% C.I. 1.87–2.26) to choose a job profile consisting of a benefits package with medical aid, pension and housing contributions worth 40% of the salary.

### Interactions by healthcare worker cadre

Interaction models (Table 5) for each of the healthcare worker cadres, revealed that preference structures across different cadres were similar, with a few exceptions. General practitioners were more likely than other cadres to select job profiles with infrequent opportunities for training (odds ratio 1.19; 95% C.I. 1.05–1.35) and were less likely than other cadres to choose a job that required them to relocate to an urban area (odds ratio 0.73; 95% C.I. 0.58–0.91), with an even stronger negative effect for relocating to a rural area (odds ratio 0.57; 95% C.I. 0.46–0.71). Pharmacy personnel were less incentivized than other cadres by a benefits package including a medical aid and pension contribution of 20% of their salary (odds ratio 0.71; 95% C.I. 0.54–0.93) compared to job profiles with no benefits. Preferences of professional nurses deviated substantially from the other cadres. Specifically, professional nurses valued opportunities for training (infrequent training odds ratio 0.88; 95% C.I. 0.78–0.99) compared to other cadres. Professional nurses were also more likely to choose a job at a public clinic than a private clinic compared to other cadres (odds ratio 0.77; 95% C.I. 0.61–0.97). Support nurses were more willing than other cadres to relocate to a rural area (odds ratio 1.77; 95% C.I. 1.41–2.23).

**Table 5:** Interaction models by healthcare worker cadre.

		**General Practitioner = 0**		**Pharmacy Personnel = 0**		**Professional Nurse = 0**		**Support Nurses = 0**	
**ATTRIBUTE**	**ATTRIBUTE LEVEL**	**Odds Ratio**	**95% C.I.**	**Odds Ratio**	**95% C.I.**	**Odds Ratio**	**95% C.I.**	**Odds Ratio**	**95% C.I.**
**WORKLOAD**	Heavy workload (vs manageable workload)	0.80**	0.75–0.85	0.77**	0.73–0.82	0.78**	0.73–0.83	0.78**	0.74–0.83
**WORKPLACE CULTURE**	Poor culture (vs enabling culture)	0.70**	0.66–0.74	0.64**	0.61–0.68	0.64**	0.60–0.68	0.65**	0.61–0.69
**EQUIPMENT**	Insufficient equipment (vs sufficient equipment)	0.66**	0.62–0.71	0.66**	0.63–0.71	0.65**	0.61–0.70	0.68**	0.64–0.72
**TRAINING**	Infrequent training (vs frequent training)	0.71**	0.66–0.75	0.75**	0.71–0.80	0.78**	0.73–0.84	0.76**	0.72–0.81
**SECTOR/ FACILITY**	Public hospital (vs public clinic)	0.88*	0.78–0.98	0.89*	0.80–0.99	0.96	0.86–1.08	0.90*	0.81–1.00
	Private clinic (vs public clinic)	0.85**	0.75–0.96	0.87*	0.78–0.99	0.97	0.85–1.11	0.88*	0.78–1.00
	Private hospital (vs public clinic)	0.94	0.83–1.05	0.98	0.88–1.09	1.04	0.92–1.17	1.01	0.91–1.13
**LOCATION**	Urban with relocation (vs urban without relocation)	0.81**	0.73–0.91	0.74**	0.67–0.83	0.72**	0.64–0.81	0.71**	0.64–0.79
	Rural without relocation (vs urban without relocation)	0.92	0.81–1.04	0.89	0.79–1.01	0.86*	0.75–0.98	0.85**	0.76–0.96
	Rural with relocation (vs urban without relocation)	0.75**	0.67–0.83	0.63**	0.57–0.70	0.62**	0.55–0.69	0.57**	0.51–0.63
**SALARY**	Salary increase of 20% (vs salary increase of 5%)	1.14*	1.02–1.27	1.07	0.97–1.18	1.05	0.94–1.18	1.11	1.00–1.23
	Salary increase of 35% (vs salary increase of 5%)	1.29**	1.14–1.47	1.30**	1.16–1.47	1.27**	1.11–1.45	1.29**	1.15–1.46
	Salary decrease to 80% of current (vs salary increase of 5%)	0.59**	0.52–0.66	0.53**	0.47–0.59	0.55**	0.49–0.62	0.53**	0.47–0.59
**BENEFITS**	Benefits–medical aid contribution of 7% of salary (vs none)	1.45**	1.30–1.62	1.41**	1.28–1.57	1.39**	1.24–1.56	1.41**	1.27–1.56
	Benefits–pension and medical aid contribution of 20% of salary (vs none)	1.83**	1.62–2.08	1.91**	1.69–2.15	1.74**	1.52–1.98	1.68**	1.49–1.90
	Benefits–pension. medical aid and housing contribution of 40% of salary (vs none)	2.06**	1.84–2.29	2.08**	1.88–2.31	2.10**	1.88–2.36	2.01**	1.81–2.22
		**General Practitioner = 1**		**Pharmacy Personnel = 1**		**Professional Nurse = 1**		**Support Nurses = 1**	
		**Odds Ratio**	**95% C.I.**	**Odds Ratio**	**95% C.I.**	**Odds Ratio**	**95% C.I.**	**Odds Ratio**	**95% C.I.**
**WORKLOAD**	Heavy workload (vs manageable workload)	0.93	0.82–1.05	1.09	0.95–1.26	1.01	0.90–1.13	0.99	0.86–1.13
**WORKPLACE CULTURE**	Poor culture (vs enabling culture)	0.79**	0.70–0.90	1.10	0.96–1.27	1.08	0.96–1.21	1.05	0.92–1.21
**EQUIPMENT**	Insufficient equipment (vs sufficient equipment)	0.99	0.88–1.13	0.99	0.86–1.13	1.07	0.95–1.20	0.91	0.80–1.05
**TRAINING**	Infrequent training (vs frequent training)	1.19**	1.05–1.35	0.97	0.85–1.12	0.88*	0.78–0.99	0.90	0.79–1.03
**SECTOR/ FACILITY**	Public hospital (vs public clinic)	1.06	0.85–1.31	1.12	0.88–1.43	0.82	0.67–1.00	1.04	0.82–1.31
	Private clinic (vs public clinic)	1.19	0.93–1.53	1.10	0.84–1.45	0.77*	0.61–0.97	1.02	0.78–1.33
	Private hospital (vs public clinic)	1.18	0.95–1.48	1.06	0.83–1.37	0.86	0.70–1.06	0.89	0.70–1.14
**LOCATION**	Urban with relocation (vs urban without relocation)	0.73**	0.58–0.91	0.98	0.77–1.25	1.13	0.92–1.38	1.29*	1.01–1.64
	Rural without relocation (vs urban without relocation)	0.85	0.66–1.08	0.92	0.70–1.22	1.08	0.86–1.36	1.18	0.90–1.55
	Rural with relocation (vs urban without relocation)	0.57**	0.46–0.71	1.00	0.79–1.27	1.08	0.88–1.31	1.77**	1.41–2.23
**SALARY**	Salary increase of 20% (vs salary increase of 5%)	0.88	0.72–1.09	1.11	0.88–1.42	1.13	0.93–1.37	0.92	0.73–1.16
	Salary increase of 35% (vs salary increase of 5%)	1.02	0.80–1.30	0.93	0.71–1.23	1.05	0.84–1.32	1.00	0.76–1.31
	Salary decrease to 80% of current (vs salary increase of 5%)	0.81	0.64–1.01	1.19	0.93–1.53	0.97	0.78–1.19	1.18	0.91–1.51
**BENEFITS**	Benefits–medical aid contribution of 7% of salary (vs none)	0.93	0.75–1.15	1.01	0.79–1.28	1.05	0.86–1.28	1.04	0.83–1.32
	Benefits–pension and medical aid contribution of 20% of salary (vs none)	0.93	0.73–1.19	0.71*	0.54–0.93	1.10	0.87–1.38	1.37*	1.04–1.79
	Benefits–pension. medical aid and housing contribution of 40% of salary (vs none)	1.04	0.84–1.29	0.95	0.74–1.20	0.93	0.77–1.14	1.18	0.93–1.49
	**Log Likelihood**	**-3973.0194**		**-4002.1752**		**-4002.3332**		**-3988.1733**	

*Significant at 95%;

**Significant at 99%

Note: Interaction terms were created by multiplying a dummy variable for each group by each attribute level and running a regression with both the original level variables and all interaction terms (e.g. for general practitioners, the dummy variable is set equal to 1 if the person is a general practitioner, and 0 for all other cadres). The top half of the table shows the odds ratios of the attribute level versus the baseline for the sample, excluding those in the specific interaction. The bottom half of the table should be interpreted for the specific cadre interaction in relation to the rest of the population, showing where preferences for each particular cadre diverge from the preferences of the rest of the sample.

**Fig 1:**
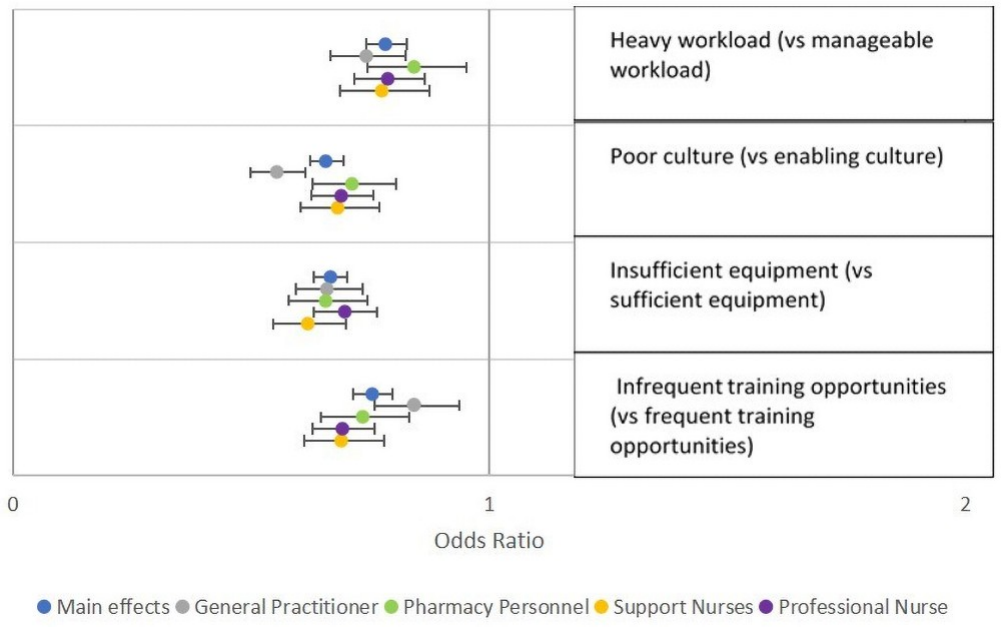
Preferences regarding workload, culture, availability of equipment and opportunities for training by healthcare worker cadre. This figure shows results of models stratified by cadre, including odds ratios and 95% confidence intervals, for attributes related to workplace conditions.

**Fig 2:**
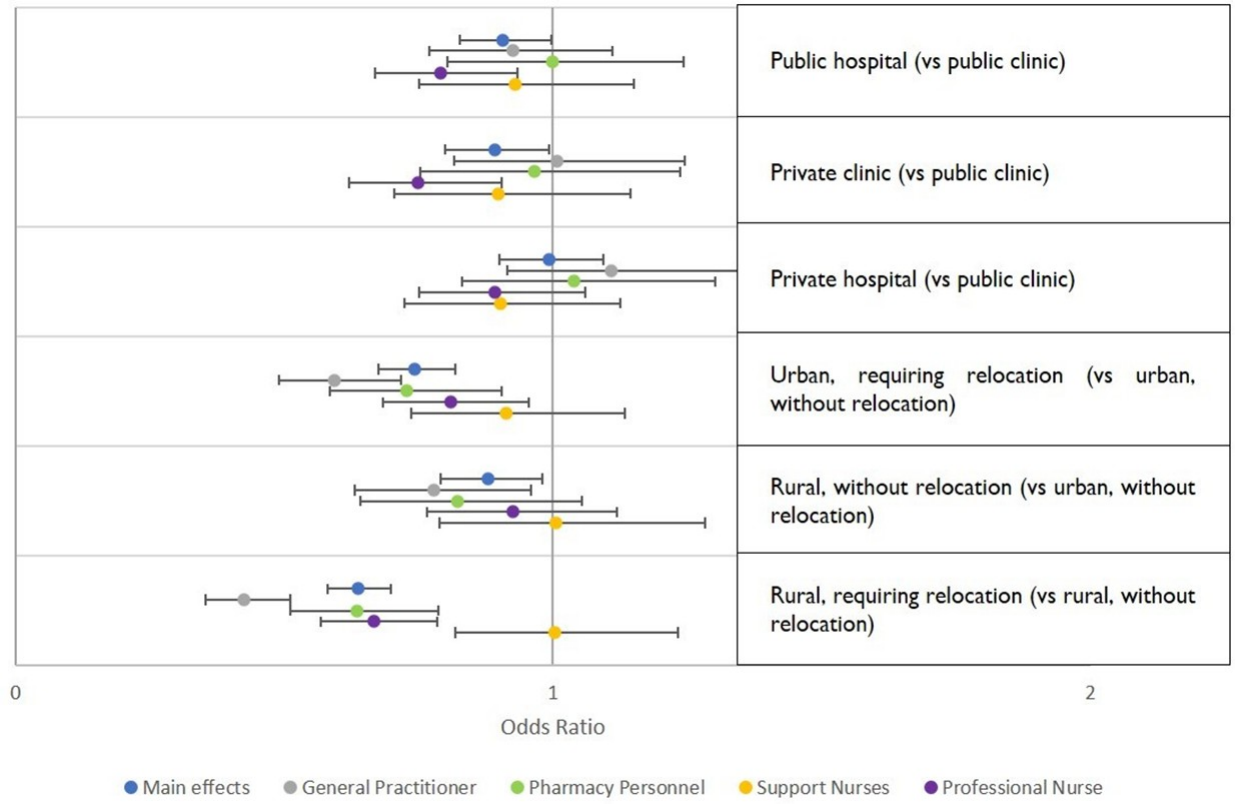
Preferences regarding sector, facility type and location by healthcare worker cadre. This figure shows results of models stratified by cadre, including odds ratios and 95% confidence intervals, for attributes related to workplace type, sector, and location.

Strong, significant effects were found for all odds ratios related to workplace conditions across all cadres (Fig 1). However, for most cadres, the sector and type of facility did not show an effect, except for professional nurses, who revealed a preference for working in a public clinic over working in a public hospital or a private clinic (Fig 2). All cadres except support nurses showed a significant negative effect for jobs requiring relocation, whether to an urban or rural location. Only general practitioners showed a preference for an urban over a rural job, when neither required relocation (Fig 2).

**Fig 3:**
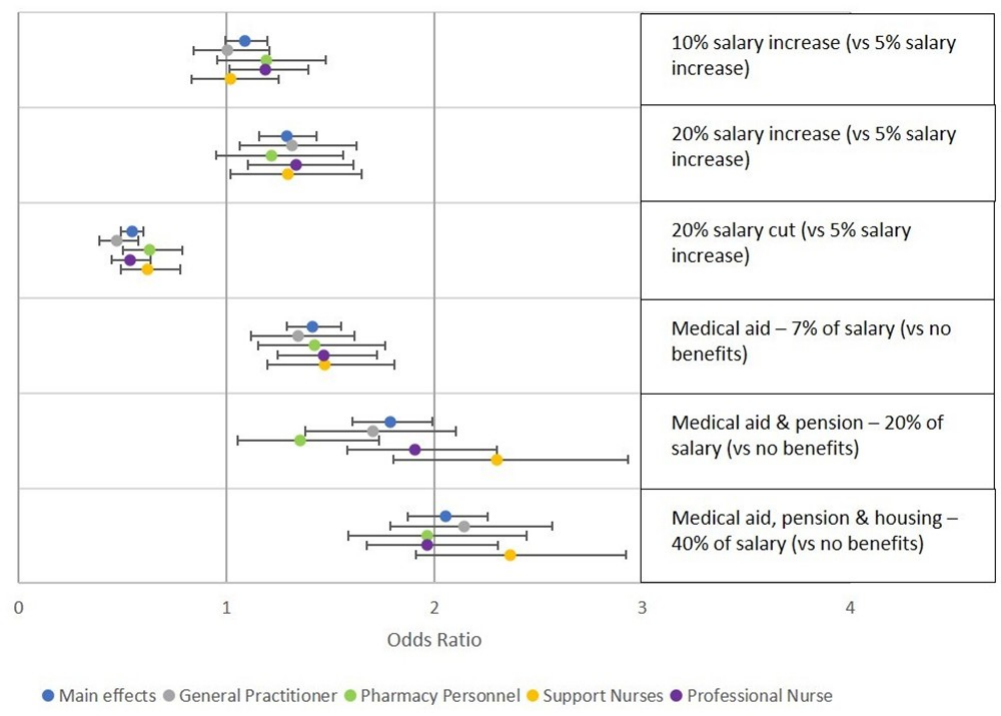
Preferences regarding salary and benefits packages. This figure shows results of models stratified by cadre, including odds ratios and 95% confidence intervals, for attributes related to salary and benefits.

Preferences related to salary and benefits did not vary significantly between healthcare worker cadres (Fig 3). Across most cadres, benefits packages showed stronger effects than salary increases. The odds of choosing a benefits package worth 20% of salary compared to no benefits package were higher than the odds of choosing a salary increase of 20% compared to a benefits package equivalent to a 7% salary increase for all cadres. Among support nurses, the odds of choosing a job alternative that included a benefits package including medical aid and pension contribution were double the odds of choosing one with no benefits package, when all other attributes were held constant.

## Discussion

In South Africa, the overwhelming majority of HIV services are delivered in the public sector. The South African government has already begun to implement strategic plans to ensure an adequate supply and distribution of healthcare workers across HIV and non-HIV health services [12, 33, 34]. This is critical to sustaining the world’s largest HIV treatment program and meet the ever-expanding communicable, non-communicable and mental healthcare needs of the population [35]. However, in both the short and long term, more will need to be done to recruit and retain public sector healthcare workers. It is important to note that some of this recruitment will happen with support from donors such as the U.S. President’s Emergency Plan for AIDS Relief (PEPFAR) and the Global Fund to Fight AIDS, Tuberculosis and Malaria (GFATM), with partners placing healthcare workers at healthcare facilities. Better understanding of what drives healthcare worker employment considerations will potentially help fill vacancies and reduce attrition. Towards this, improving the working conditions is crucial to attract and retain appropriate cadres to the public sector, especially in underserved areas.

Our findings suggest that workload, workplace culture, availability of equipment and opportunities for training significantly drive job preferences, which is in line with previous research [7, 9, 15, 21]. Of particular note, we found that preferences regarding employment sector (public vs. private) itself was a weak driver of choice. Moreover, the type of facility (clinic vs. hospital) also showed a small effect on preferences. Together, these results suggest that migration of healthcare workers between sectors and different types of facilities is largely influenced by other job-related factors and that overall, individuals were willing to make trade-offs to work in public or rural facilities, provided the working conditions were good (including a good workplace culture, manageable workload, good opportunities for training and development, and well equipped facilities).

Previous work has shown that recruiting healthcare workers to and retaining them in rural posts can be difficult [7, 15, 36, 37]. However, our work strongly suggests that having to relocate is more influential than the location itself in driving employment decisions, especially for the nursing cadres. Ultimately, in comparison to the other organizational, infrastructural and remuneration considerations, the location of a health facility did not, in isolation, play a significant role in determining preferences. However, as reported previously, rural posts in South Africa often retain those characteristics that negatively affect employment considerations of healthcare workers, including ill-equipped and dilapidated facilities, high patient loads, inadequate staffing, and a lack of access to training and developmental opportunities [9, 38]. If these conditions persist, recruiting healthcare workers to rural sites will prove challenging given the added resistance to relocate. Finally, understanding how socio-demographic factors and relocation preferences interact in the context of overall preference structures could assist in the development of initiatives aimed at recruitment to public sector posts in rural communities.

As reported in previous studies [7, 9, 15], we found remuneration to have an effect on preferences across healthcare worker cadres, although modest salary increases of 5 or 10% did not significantly alter preferences. Study respondents were significantly more likely to select job profiles which contained improved benefits packages compared to a ‘no benefits’ baseline scenario. Preferences were stronger for benefits package worth 20% of salary than a salary increase of 20% across all cadres. This indicates that though benefits packages and salary increases both have monetary value, they are perceived differently by healthcare workers. Although at a first glance, this may appear to contradict economic theory, we believe this may not be the case, as benefits packages carry other potentially utility generating characteristics, a finding consistent with previous research [39]. There may be a recognition, for example, that employers have a comparative advantage in purchasing benefits (such as health insurance or pension instruments) relative to employees, and thus additional benefits would be more appealing than a higher salary [40]. Since certain benefits are tax exempt, individuals may also perceive some benefits as worth more than salary, even if they have the same real value. Benefits packages may be more attractive because they are a form of “forced savings”, which may be appealing to individuals who share their income with their families and are unlikely to be able to ring-fence as effectively if the additional remuneration came in the form of money. Participants in this study showed that they were willing to make trade-offs between increased remuneration and working conditions (including workplace culture, opportunities for training, well equipped facilities and workloads), but only for relatively substantial increases in remuneration. In the long run, it may be more cost effective to improve working conditions at rural and public facilities rather than using monetary incentives, but in the short run, benefits packages can be a useful recruitment strategy. Additional economic evaluations of these options should be considered in future research.

While we did not find a reluctance to work in the public sector, South Africa’s two-tiered health sector presents options for healthcare workers. If public sector facilities are associated with job factors that individuals want to avoid, like heavy workload and poor infrastructure, they may be more likely to choose employment in the private sector. This presents a significant challenge for policy makers trying to ensure an efficient and equitable distribution of healthcare workers. Because remuneration packages and working conditions are often better in the private sector, particularly for general practitioners and specialists, it will be difficult to recruit and retain these cadres in the public sector. Although private and public sector nurses may not make substantially different salaries, the increased availability of resources in the private sector often results in substantially better working conditions [11].

Further analysis of the preference structures of those who recently moved sectors could provide a more nuanced understanding of our findings and highlight the biggest motivating factors behind their decision. Additionally, it is important to note that postings with desirable attributes (i.e. generous benefits packages, higher salaries, and above average working conditions) are likely in high demand. An analysis of the supply and demand of health posts may help identify where potential shortages are likely to occur. Potential shortages of specific cadres could be avoided by tailoring posts to their preferences. For example, the government could partner with educational institutions that have strong online content and provide nurses access to the basic resources (i.e., laptop computer, internet access and data) needed to access the training opportunities that they are seeking.

The results of this DCE suggest that there are important differences in preference structures between cadres. Specifically, general practitioners and pharmacy personnel showed a stronger aversion to having to relocate than nursing cadres. Although our sample is not necessarily representative of the entire South African health workforce, our results suggest that certain strategies may improve recruitment and retention to underserved areas. These include recruiting students to or from these areas by offering bursaries and additional incentives once they have finished their training, which is already implemented in some South African settings [9]. Ensuring that salary and benefits packages are well-designed will be an important component of this strategy; however, ensuring that public sector working conditions and operational infrastructure are improved will remain a vital component of any recruitment and retention initiatives.

This study is in response to the only systematic review on the use of discrete choice experiments to inform health workforce policy [17]. The review concluded that there was a need to include a broader range of health cadres, with previous studies focusing primarily on doctors or medical students. This study, whilst focusing on South African health workers, is powered to allow for stratified analysis by health cadre and is the first South African DCE to include an examination of the preference structures of pharmacists and pharmacy assistants. By recruiting participants working from both the public and private health sectors and in rural and urban areas, we were able to constitute a sample of participants who could draw on their diverse experiences to better understand employment choices.

One potential study limitation was that nearly half of participants opted to complete the DCE remotely via an online survey platform. Although this improved recruitment, particularly among general practitioners, we were initially concerned that those responding electronically may have had a different understanding of attributes and levels than those participants whose questionnaire was administered by study staff. Completion rates amongst participants who responded online were high and an analysis to check for systematic differences between those who responded online versus in-person with study staff showed no evidence of bias, suggesting that online delivery of DCE surveys to healthcare professionals can generate good quality data. This study is also limited in its generalizability to all areas of South Africa because only three provinces were included for sampling.

## Conclusions

Our findings regarding employment preferences can help guide the formulation of strategies aimed at attracting and retaining the healthcare workers who provide critical services within South Africa’s public health system, including its HIV care and treatment program. As new healthcare workers are hired to meet the demand for services, especially for HIV, the sustainability of these positions needs to be ensured by addressing crucial workplace conditions and fiscal space. For staff initially supported by donors such as PEPFAR and GFATM, it is vital that these individuals can be transitioned to government’s wage bill and retained through strategies that support preferences. As expected, salary was an important consideration across all cadres. However, benefits packages had an even greater impact on overall preferences. Offering an attractive benefits package is likely to be able to partially offset the negative effects of other attributes that may engender pursuit of alternative employment opportunities. Considerations regarding working conditions–workload, workplace culture, the quality and availability of equipment, and opportunities for training and development–formed an equally important part of preference structures across all cadres. Facilities in the public sector should prioritize the development of effective operational systems, allowing for better infrastructure and asset management, to ensure continuous supply of equipment and resources. Health departments also need to strengthen their internal workplace skills plans to monitor skills shortages and identify training and development opportunities for healthcare workers. Preference differences across sectors and facility types were less important, although this varied across cadres. Though there was a small preference for urban compared to rural postings, having to relocate had a much larger effect on preferences. Crucially, our evidence suggests that factors amenable to improvement such as workplace conditions and remuneration packages have a greater influence on employment decisions than employment sector or location.

## Supporting information

S1 File DCE questionnaire version 1. (PDF)

S2 File DCE questionnaire version 2. (PDF)

S3 File DCE questionnaire version 3. (PDF)

S4 File DCE questionnaire version 4. (PDF)
