# Supplementary material for: Employment preferences of healthcare workers in South Africa: Findings from a discrete choice experiment
Source: PLoS One. 2021 Apr 28;16(4):e0250652. doi: 10.1371/journal.pone.0250652 (PMC8081228; doi:10.1371/journal.pone.0250652)
Supplement: S4 File — (PDF) [file pone.0250652.s004.pdf]

DCE Questionnaire

Version 4

Question 4.1

OPTION A		OPTION B	
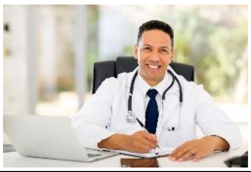	You have a manageable workload	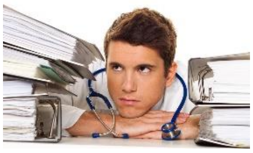	You have a heavy workload
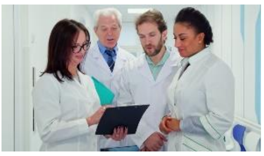	You work in an good culture	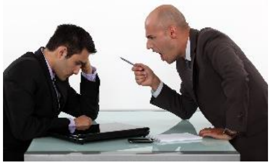	You work in a poor culture
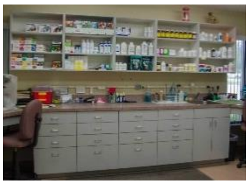	There is a sufficient availability of equipment and drugs at your work place	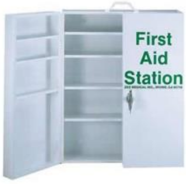	There is an insufficient availability of equipment and drugs at your work place
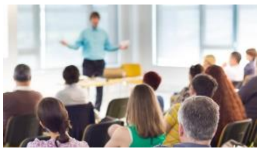	There are frequent and sufficient opportunities for training, personal development, and continuing education at your work place	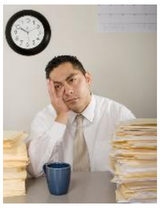	There are infrequent or minimal opportunities for training, personal development, and continuing education at your work place
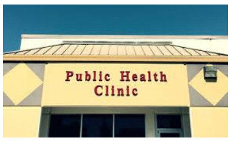	You work at a public clinic	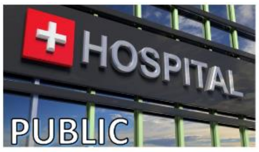	You work at a public hospital
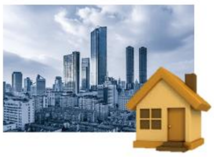	In or near a town or city that doesn't require you to relocate	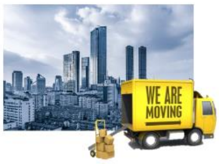	In or near a town or city that requires you to relocate
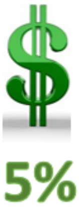	You receive a 5% increase on your current salary	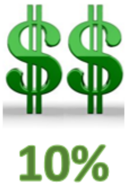	You receive a 10% increase on your current salary
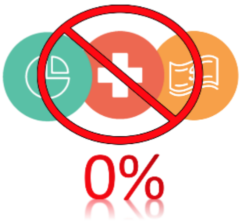	You receive no employee benefits or allowances	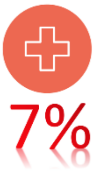	You receive a medical aid contribution worth 7% of your salary

Question 4.2

**OPTION A**		**OPTION B**	
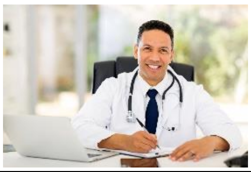	You have a manageable workload	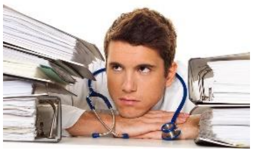	You have a heavy workload
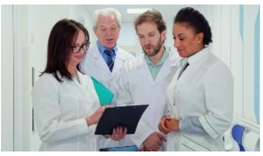	You work in an good culture	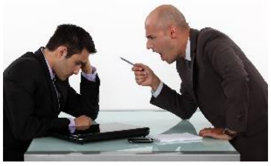	You work in a poor culture
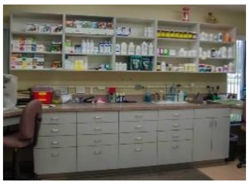	There is a sufficient availability of equipment and drugs at your work place	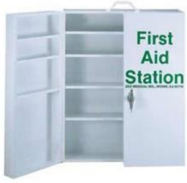	There is an insufficient availability of equipment and drugs at your work place
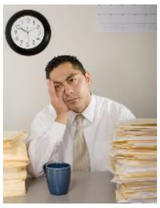	There are infrequent or minimal opportunities for training, personal development, and continuing education at your work place	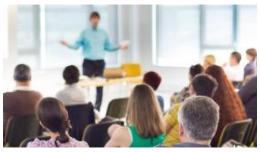	There are frequent and sufficient opportunities for training, personal development, and continuing education at your work place
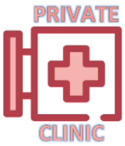	You work at a private clinic	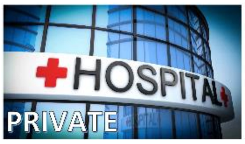	You work at a private hospital
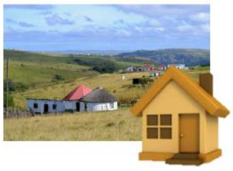	In a rural community outside of towns or cities that doesn't require you to relocate	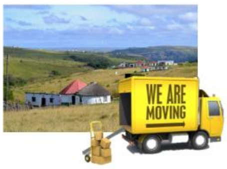	In a rural community outside of towns or cities that requires you to relocate
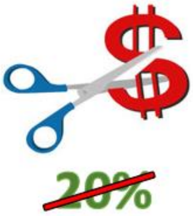	You receive a salary cut of 20% from your current salary	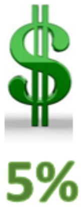	You receive a 5% increase on your current salary
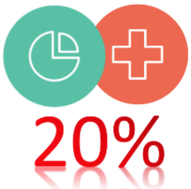	You receive a pension and medical aid contribution worth 20% of your salary	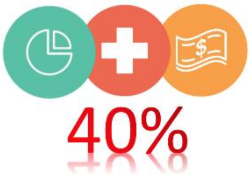	You receive a pension, medical aid contribution, and a living allowance worth 40% of your salary

Question 4.3

OPTION A		OPTION B	
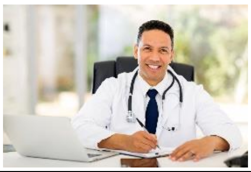	You have a manageable workload	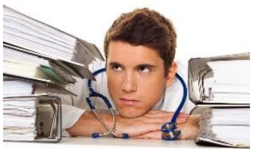	You have a heavy workload
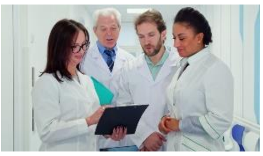	You work in an good culture	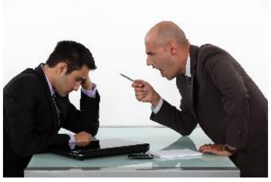	You work in a poor culture
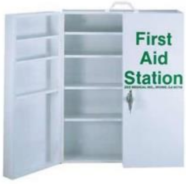	There is an insufficient availability of equipment and drugs at your work place	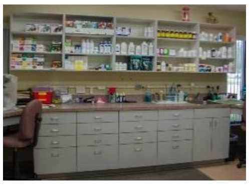	There is a sufficient availability of equipment and drugs at your work place
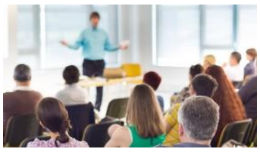	There are frequent and sufficient opportunities for training, personal development, and continuing education at your work place	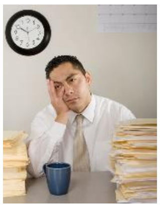	There are infrequent or minimal opportunities for training, personal development, and continuing education at your work place
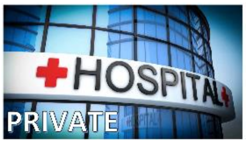	You work at a private hospital	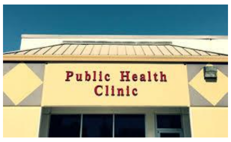	You work at a public clinic
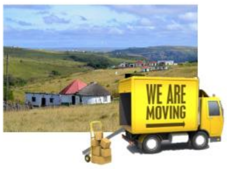	In a rural community outside of towns or cities that requires you to relocate	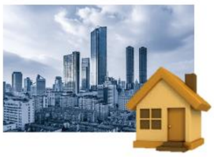	In or near a town or city that doesn't require you to relocate
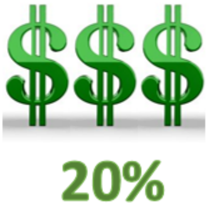	You receive a 20% increase on your current salary	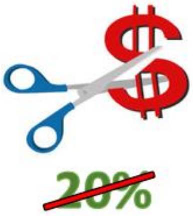	You receive a salary cut of 20% from your current salary
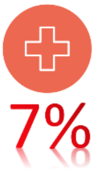	You receive a medical aid contribution worth 7% of your salary	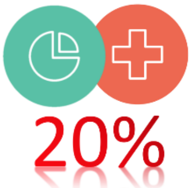	You receive a pension and medical aid contribution worth 20% of your salary

Question 4.4

OPTION A		OPTION B	
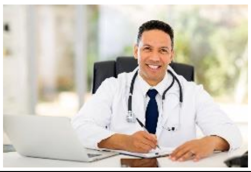	You have a manageable workload	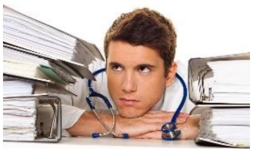	You have a heavy workload
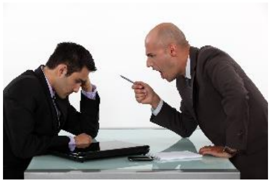	You work in a poor culture	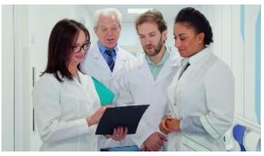	You work in an good culture
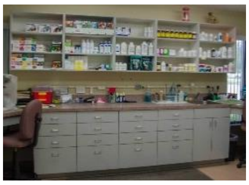	There is a sufficient availability of equipment and drugs at your work place	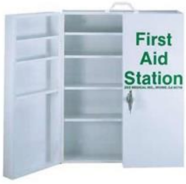	There is an insufficient availability of equipment and drugs at your work place
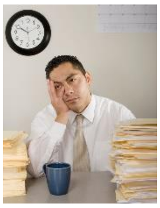	There are infrequent or minimal opportunities for training, personal development, and continuing education at your work place	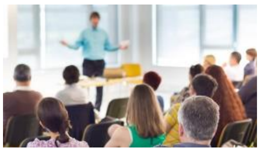	There are frequent and sufficient opportunities for training, personal development, and continuing education at your work place
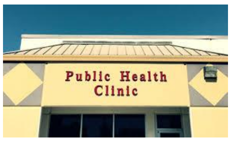	You work at a public clinic	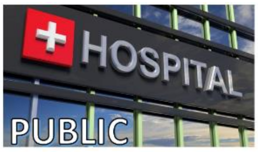	You work at a public hospital
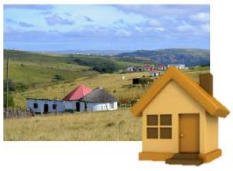	In a rural community outside of towns or cities that doesn't require you to relocate	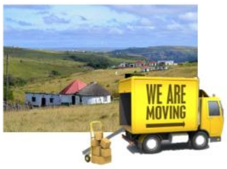	In a rural community outside of towns or cities that requires you to relocate
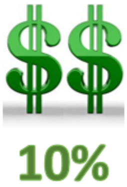	You receive a 10% increase on your current salary	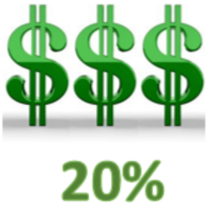	You receive a 20% increase on your current salary
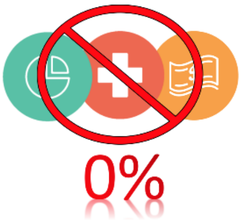	You receive no employee benefits or allowances	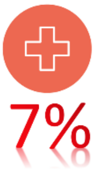	You receive a medical aid contribution worth 7% of your salary

Question 4.5

OPTION A		OPTION B	
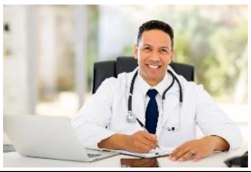	You have a manageable workload	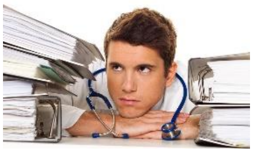	You have a heavy workload
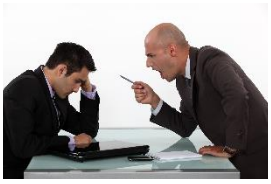	You work in a poor culture	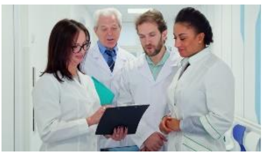	You work in an good culture
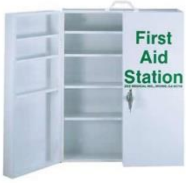	There is an insufficient availability of equipment and drugs at your work place	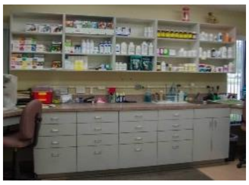	There is a sufficient availability of equipment and drugs at your work place
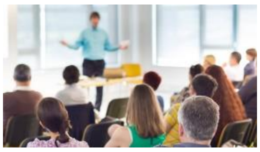	There are frequent and sufficient opportunities for training, personal development, and continuing education at your work place	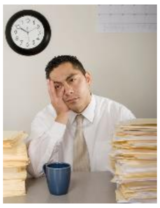	There are infrequent or minimal opportunities for training, personal development, and continuing education at your work place
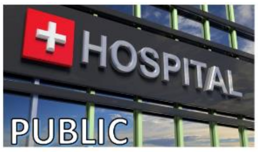	You work at a public hospital	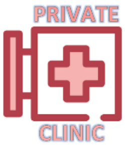	You work at a private clinic
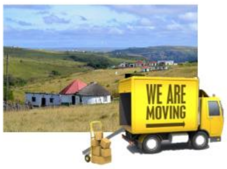	In a rural community outside of towns or cities that requires you to relocate	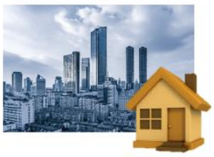	In or near a town or city that doesn't require you to relocate
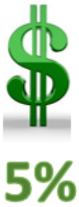	You receive a 5% increase on your current salary	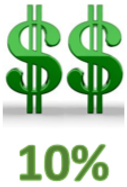	You receive a 10% increase on your current salary
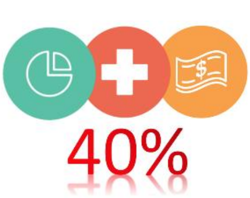	You receive a pension, medical aid contribution, and a living allowance worth 40% of your salary	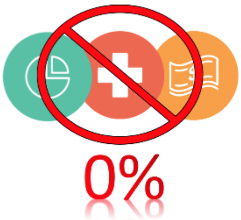	You receive no employee benefits or allowances

Question 4.6

**OPTION A**		**OPTION B**	
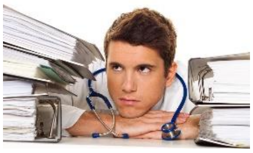	You have a heavy workload	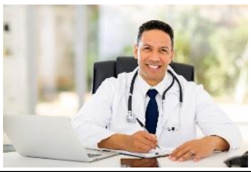	You have a manageable workload
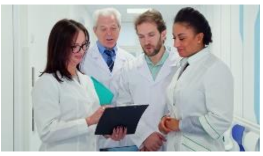	You work in an good culture	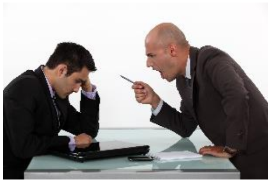	You work in a poor culture
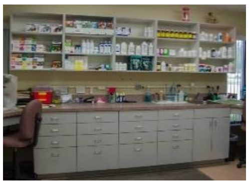	There is a sufficient availability of equipment and drugs at your work place	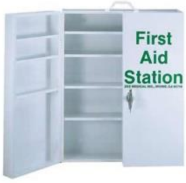	There is an insufficient availability of equipment and drugs at your work place
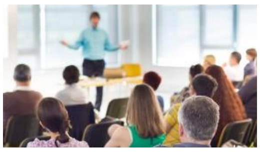	There are frequent and sufficient opportunities for training, personal development, and continuing education at your work place	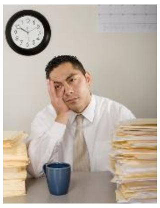	There are infrequent or minimal opportunities for training, personal development, and continuing education at your work place
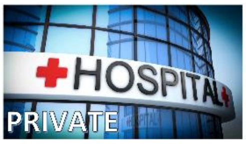	You work at a private hospital	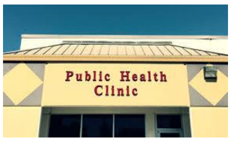	You work at a public clinic
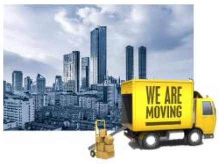	In or near a town or city that requires you to relocate	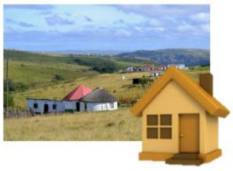	In a rural community outside of towns or cities that doesn't require you to relocate
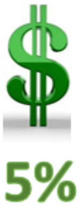	You receive a 5% increase on your current salary	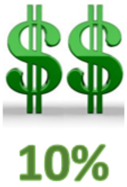	You receive a 10% increase on your current salary
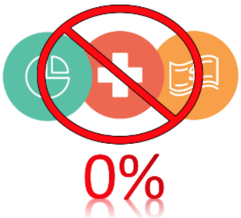	You receive no employee benefits or allowances	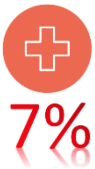	You receive a medical aid contribution worth 7% of your salary

Question 4.7

OPTION A		OPTION B	
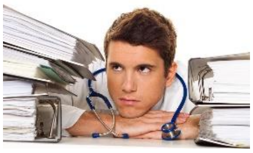	You have a heavy workload	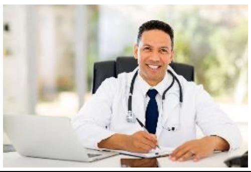	You have a manageable workload
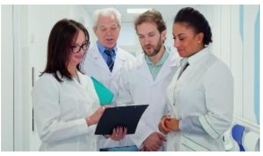	You work in an good culture	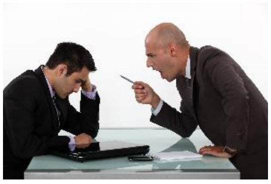	You work in a poor culture
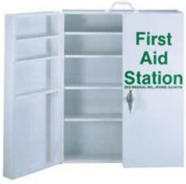	There is an insufficient availability of equipment and drugs at your work place	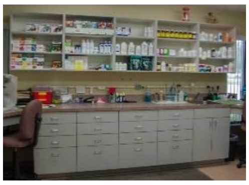	There is a sufficient availability of equipment and drugs at your work place
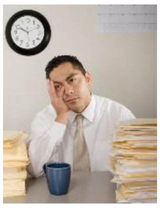	There are infrequent or minimal opportunities for training, personal development, and continuing education at your work place	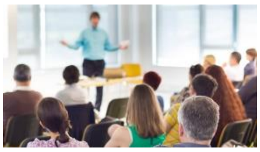	There are frequent and sufficient opportunities for training, personal development, and continuing education at your work place
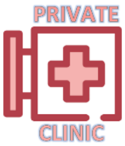	You work at a private clinic	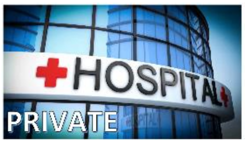	You work at a private hospital
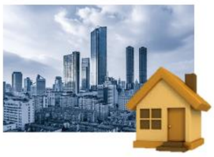	In or near a town or city that doesn't require you to relocate	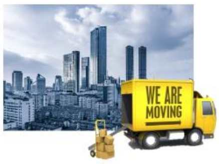	In or near a town or city that requires you to relocate
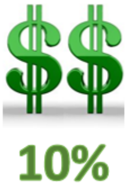	You receive a 10% increase on your current salary	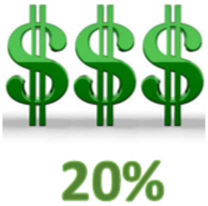	You receive a 20% increase on your current salary
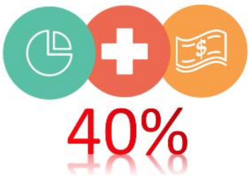	You receive a pension, medical aid contribution, and a living allowance worth 40% of your salary	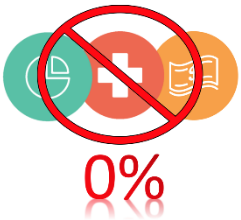	You receive no employee benefits or allowances

Question 4.8

**OPTION A**		**OPTION B**	
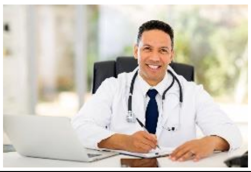	You have a manageable workload	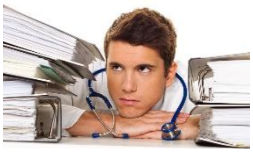	You have a heavy workload
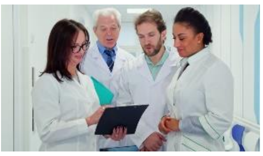	You work in an good culture	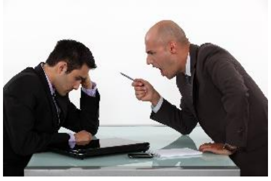	You work in a poor culture
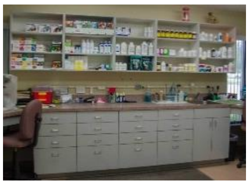	There is a sufficient availability of equipment and drugs at your work place	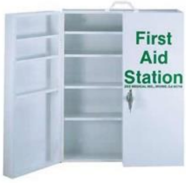	There is an insufficient availability of equipment and drugs at your work place
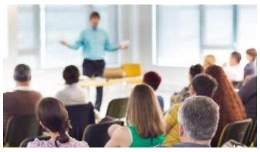	There are frequent and sufficient opportunities for training, personal development, and continuing education at your work place	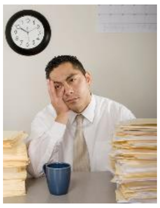	There are infrequent or minimal opportunities for training, personal development, and continuing education at your work place
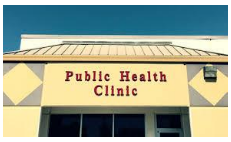	You work at a public clinic	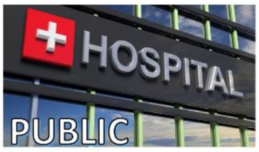	You work at a public hospital
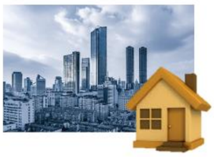	In or near a town or city that doesn't require you to relocate	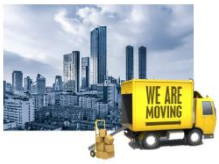	In or near a town or city that requires you to relocate
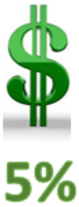	You receive a 5% increase on your current salary	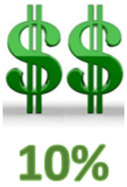	You receive a 10% increase on your current salary
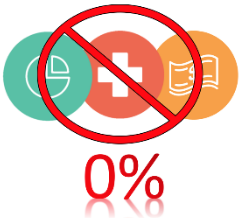	You receive no employee benefits or allowances	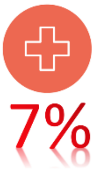	You receive a medical aid contribution worth 7% of your salary

Question 4.9

OPTION A		OPTION B	
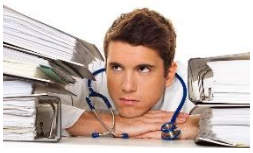	You have a heavy workload	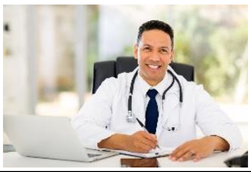	You have a manageable workload
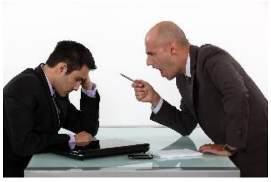	You work in a poor culture	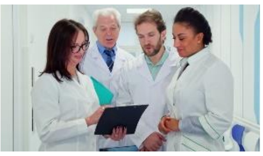	You work in an good culture
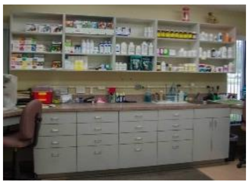	There is a sufficient availability of equipment and drugs at your work place	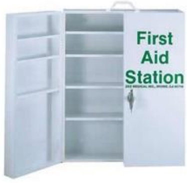	There is an insufficient availability of equipment and drugs at your work place
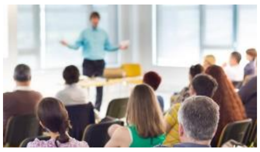	There are frequent and sufficient opportunities for training, personal development, and continuing education at your work place	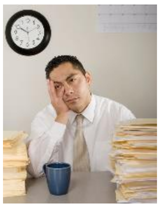	There are infrequent or minimal opportunities for training, personal development, and continuing education at your work place
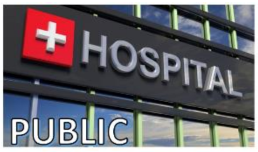	You work at a public hospital	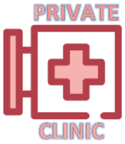	You work at a private clinic
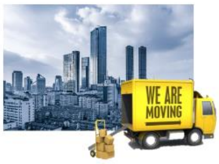	In or near a town or city that requires you to relocate	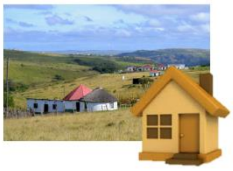	In a rural community outside of towns or cities that doesn't require you to relocate
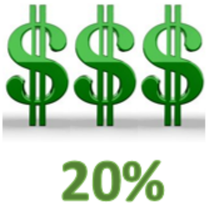	You receive a 20% increase on your current salary	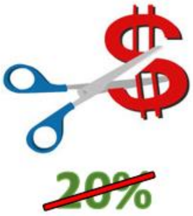	You receive a salary cut of 20% from your current salary
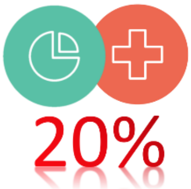	You receive a pension and medical aid contribution worth 20% of your salary	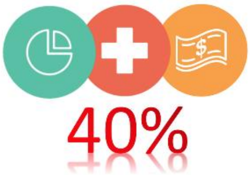	You receive a pension, medical aid contribution, and a living allowance worth 40% of your salary

Question 4.10

OPTION A		OPTION B	
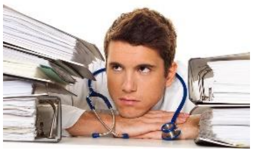	You have a heavy workload	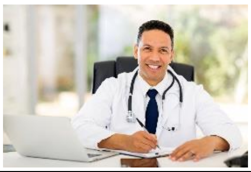	You have a manageable workload
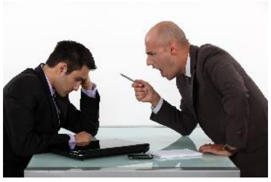	You work in a poor culture	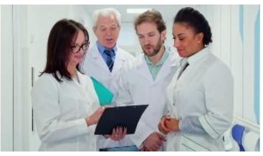	You work in an good culture
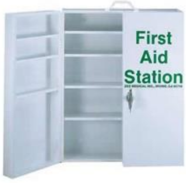	There is an insufficient availability of equipment and drugs at your work place	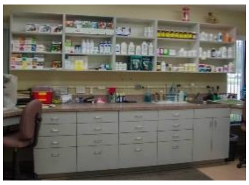	There is a sufficient availability of equipment and drugs at your work place
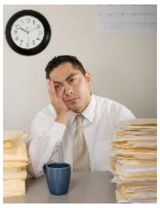	There are infrequent or minimal opportunities for training, personal development, and continuing education at your work place	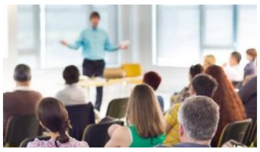	There are frequent and sufficient opportunities for training, personal development, and continuing education at your work place
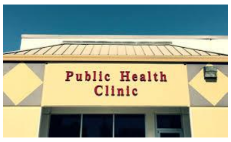	You work at a public clinic	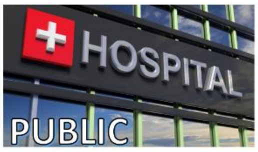	You work at a public hospital
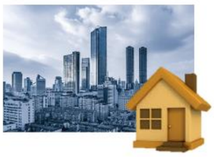	In or near a town or city that doesn't require you to relocate	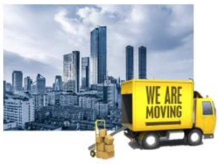	In or near a town or city that requires you to relocate
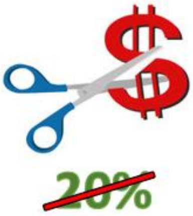	You receive a salary cut of 20% from your current salary	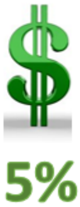	You receive a 5% increase on your current salary
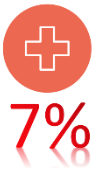	You receive a medical aid contribution worth 7% of your salary	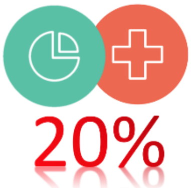	You receive a pension and medical aid contribution worth 20% of your salary
